# Deep Femoral Artery Aneurysm Resection and Revascularization With a Saphenous Vein

**DOI:** 10.7759/cureus.44092

**Published:** 2023-08-25

**Authors:** Hideki Sasaki, Yoshiaki Sone, Yukihide Numata, Shinji Kamiya, Syunta Hayakawa

**Affiliations:** 1 Cardiovascular Surgery, Nagoya City University East Medical Center, Nagoya, JPN

**Keywords:** lateral circumflex femoral artery, revascularization, saphenous vein, computed tomography, deep femoral artery aneurysm

## Abstract

An incidental discovery was made of a right deep femoral artery aneurysm (DFAA) in a plain computed tomography (CT) scan of a 72-year-old male. Although he had been diagnosed with type B aortic dissection six years ago and was followed for 12 months in the outpatient clinic, the patient was no longer receiving regular checkups. After a thorough discussion between cardiovascular surgeons and interventional radiologists, it was decided to proceed with aneurysm resection and revascularization. The postoperative course was uneventful, and the patient was discharged home without complications.

## Introduction

Although deep femoral artery aneurysm (DFAA) is a rare and potentially life-threatening condition, patients are usually asymptomatic unless its diameter is rather large or DFAA ruptures [1-3]. When encountering a patient with DFAA, it is important to choose the appropriate procedure. In the current era, there are treatment options for this kind of patient such as aneurysm resection, aneurysm resection with revascularization, and endovascular surgery [1]. We present a case in which aneurysm resection with revascularization was performed.

## Case presentation

**Figure 1:**
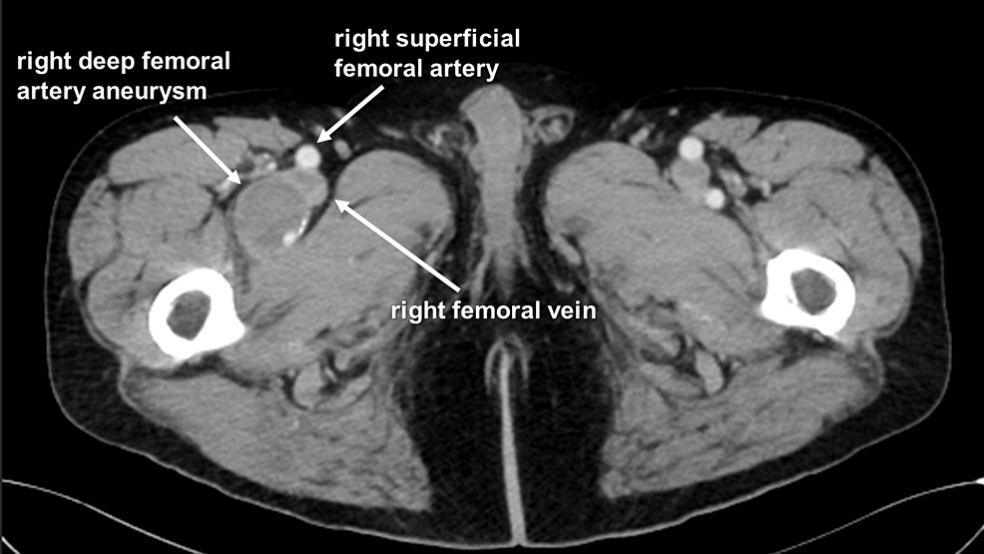
Preoperative contrast-enhanced CT Contrast-enhanced CT revealing right deep femoral artery aneurysm CT: computed tomography

A 72-year-old male was referred to the cardiovascular department for the diagnosis of a right DFAA. The patient’s medical history was complicated by a type B aortic dissection six years ago and the recent onset of myelodysplasia syndrome (MDS). However, the patient dropped out from the regular surveillance of the aortic dissection. An attending physician in the internal medicine department ordered a computed tomography (CT) scan, which revealed a right DFAA with a diameter of 3.5×3 cm (Figure 1).

**Figure 2:**
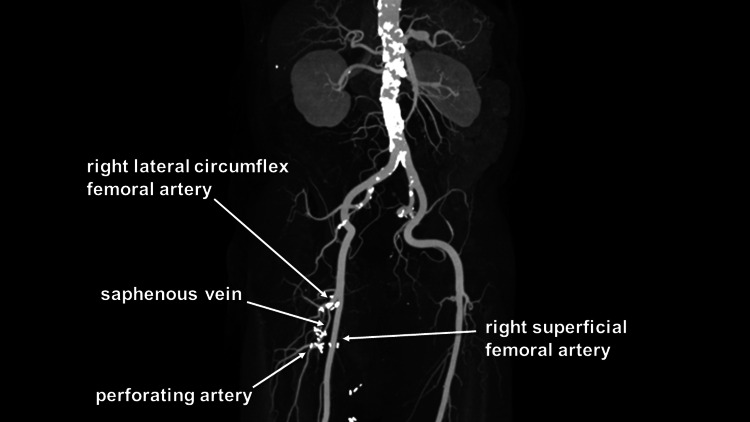
Postoperative contrast-enhanced CT Contrast-enhanced CT revealing a great saphenous vein anastomosed between the right lateral circumflex femoral artery and the perforating artery CT: computed tomography

After a thorough discussion between cardiovascular surgeons and interventional radiologists, it was decided to perform an aneurysm resection with revascularization using a saphenous vein. Under general anesthesia, the patient was placed in the supine position. The right DFAA, the right lateral circumflex femoral artery (RLCFA), and the perforating artery were exposed and secured. A great saphenous vein (GSV) was harvested. After clamping the inflow and outflow arteries, the aneurysm was incised, and the thrombus was evacuated. The distal anastomosis site was created at the perforating artery, and the distal end of the GSV was anastomosed to it. The proximal end of the GSV was anastomosed to the RLCFA. Postoperative contrast-enhanced CT showed that the GSV remained patent (Figure 2). The patient was discharged home on the sixth postoperative day.

## Discussion

DFAA is rare and accounts for 0.5% of all peripheral artery aneurysms [1]. While the pseudoaneurysm of the deep femoral artery can occur after invasive procedures [4]​​​​​, degenerative DFAA is even rarer in clinical settings. Patients are typically asymptomatic, as in the current case, unless the diameter of the aneurysm is relatively large or DFAA ruptures [2,3]. The choice of treatment was based on the specifics of each individual case. There are three points to discuss in the current case. The first is the etiology, the second is the diagnostic tool, and the third is therapeutic options.

The first point is that the etiology of DFAA in the current case is unknown. Although a type B aortic dissection occurred six years ago, a femoral aneurysm was not detected. No catheter was inserted at that time. No traumatic events have been noted since then. While azacitidine was prescribed for MDS, it is not clear whether it had an effect on the femoral aneurysm. In the literature, contralateral deep femoral artery aneurysms, as well as other aneurysms such as abdominal aortic, iliac artery, and popliteal artery aneurysms, have been noted to occur at the presentation of DFAA [1]. Among these, abdominal aortic aneurysms are often found. Additionally, the systematic review included 18 case reports and a cadaver study, encompassing a total of 27 cases of DFAA. Ruptures occurred in 18.5% of these cases. Considering that the patient’s CT showed an atherosclerotic change from the aortic arch to the iliac arteries, it may be valid to consider atherosclerosis as the main causative factor.

The second point to consider is the diagnostic tools. The majority of DFAA patients are asymptomatic, making it unusual to detect in the early stages. Possible symptoms include pain, swelling, and a pulsatile mass when it becomes large in size. In the current case, the patient had been undergoing CT scans in the outpatient clinic for type B aortic dissection for 12 months; however, the patient was eventually dropped from the follow-up. When the attending physician from the internal medicine department ordered a CT scan to address the aorta, deep femoral artery aneurysm was incidentally detected. Although ultrasound was used in 33.3% of cases and magnetic resonance angiography (MRA) in 7.4% of cases for diagnostic purposes, computed tomography angiography (CTA) remains the most frequently used modality (85.2%) [1]. Considering the patient’s medical history of type B aortic dissection, it is valid to order CT in the current case.

The third point to discuss is the selection of the procedures. There are three therapeutic options to treat DFAA: simultaneous aneurysm resection with revascularization (56.1%), ligation or resection without revascularization (28.0%), and endovascular embolization (9.8%) [1]. When collateral vessels perfuse the distal deep femoral artery, revascularization may not be necessary. In such cases, the orifice of the perforating artery can be closed from the inside after opening the aneurysm [5]. Transcatheter endovascular coil embolization is a valuable approach that can minimize the invasiveness of the procedure since the dissection of the perforating artery can sometimes be challenging. Engaging the catheter into the perforating artery can sometimes be challenging. Moreover, this difficulty is exacerbated in cases of rupture. In such situations, opting for deep femoral artery ligation without revascularization can help control antegrade flow into DFAA. However, it is important to note that backflow from the residual perforating artery can potentially lead to the recurrence of aneurysms, including daughter aneurysms. Hamamoto et al. reported a redo case in which direct percutaneous puncture embolization with N-butyl-cyanoacrylate was used to treat DFAA [6]. However, in cases where the revascularization of the perforating artery is necessary to prevent lower limb ischemia, surgeons should expose and secure it. The selection of procedures can be influenced by factors such as the size and anatomical position of the aneurysm, the location of the perforating artery, and the degree of atherosclerosis from the iliac artery to the superficial femoral artery. In the systematic review, a saphenous vein is often used compared to a prosthetic graft when revascularization is chosen [1]. In the current case, we opted for aneurysm resection and revascularization using a saphenous vein. Although significant stenosis was not found from the right iliac artery to the right superficial femoral artery, calcification was noted. This calcification might lead to significant stenosis in these areas in the near future. Additionally, the distal perforating artery was not too deep to revascularize. A thorough preoperative CTA-based discussion is of utmost importance.

## Conclusions

DFAA is rare and asymptomatic. However, potential patients might be present in an aging society, considering that the etiology is mostly atherosclerosis. When a patient has a medical history of aortic-related diseases, DFAA might be detected in a CT scan.

The management of the distal perforating artery is crucial in deciding the operative procedure. It is sometimes positioned too deep for revascularization. Given this situation, surgeons need to decide whether the perforating artery should be revascularized during preoperative discussions. When feasible, revascularization using a saphenous vein is a valid option to prevent lower limb ischemia. Elaborating on a plan based on CTA and ensuring timely intervention are important to achieve desirable results.
